# Global selection of *Plasmodium falciparum* virulence antigen expression by host antibodies

**DOI:** 10.1038/srep19882

**Published:** 2016-01-25

**Authors:** Abdirahman I. Abdi, George M. Warimwe, Michelle K. Muthui, Cheryl A. Kivisi, Esther W. Kiragu, Gregory W. Fegan, Peter C. Bull

**Affiliations:** 1 KEMRI-Wellcome Trust Research Programme, P.O. Box 230-80108, Kilifi, Kenya; 2 Department of Biochemistry and Chemistry, Pwani University, P.O. Box 195, 80108, Kilifi, Kenya; 3 Nuffield Department of Clinical Medicine, John Radcliffe Hospital, University of Oxford, Oxford, OX3; 4 The Jenner Institute, University of Oxford, ORCRB, Roosevelt Drive, Oxford, OX3 7DQ, UK; 5 Centre for Research in Therapeutic Sciences and, Institute for Healthcare Management, Strathmore University, P.O. Box 59857-00200 Nairobi, Kenya

## Abstract

Parasite proteins called PfEMP1 that are inserted on the surface of infected erythrocytes, play a key role in the severe pathology associated with infection by the *Plasmodium falciparum* malaria parasite. These proteins mediate binding of infected cells to the endothelial lining of blood vessels as a strategy to avoid clearance by the spleen and are major targets of naturally acquired immunity. PfEMP1 is encoded by a large multi-gene family called *var*. Mutually-exclusive transcriptional switching between *var* genes allows parasites to escape host antibodies. This study examined in detail the patterns of expression of *var* in a well-characterized sample of parasites from Kenyan Children. Instead of observing clear inverse relationships between the expression of broad sub-classes of PfEMP1, we found that expression of different PfEMP1 groups vary relatively independently. Parasite adaptation to host antibodies also appears to involve a general reduction in detectable var gene expression. We suggest that parasites switch both between different PfEMP1 variants and between high and low expression states. Such a strategy could provide a means of avoiding immunological detection and promoting survival under high levels of host immunity.

*Plasmodium falciparum* erythrocyte membrane protein 1 (PfEMP1) is a diverse family of proteins that are inserted into the surface of *P. falciparum* infected erythrocytes (IE). PfEMP1 plays an important role in malaria pathology by mediating cytoadhesion of parasite IE to various host receptors including CD36^1^,^2^, Intercellular adhesion molecule–1(ICAM-1^3^), endothelial protein C receptor (EPCR)^4^ on the vascular endothelial cells and complement receptor (CR1)^5^,^6^ on erythrocytes^7^,^8^. Cytoadhesion of IE in the deep microvasculature allows parasites to avoid passage through the spleen where they would normally be removed from circulation^9^. When the parasite burden is high, this parasite survival strategy causes vascular occlusion contributing to the impaired perfusion thought to be the main cause of the distinct pathology associated with infections by this species of malaria parasite^10^.

PfEMP1 is encoded by a multi-gene family called *var*^11^,^12^,^13^. Each parasite genome contains approximately 60 *var* genes, which are expressed in a mutually exclusive manner^14^. Switches in *var* gene expression allow the parasite to evade host immunity and prolong infection by evading antibody response^13^,^15^,^16^. Parasites that survive within the host are those that express PfEMP1 variants corresponding to gaps in the endogenous repertoire of host antibodies^17^,^18^.

Despite their immense molecular diversity, mainly generated through recombination events^19^,^20^,^21^,^22^,^23^,^24^, *var genes* can be classified into three major groups, A, B, and C according to sequence features found in their 5′ un-translated region^14^,^25^. These groups are broadly associated with the structural organization and size of the protein, with group A PfEMP1 tending to be longer than non-group A PfEMP1^26^. Recently a functional classification based on the presence of commonly occurring combinations of specific Duffy binding-like (DBL) and cysteine-rich inter-domain region (CIDR) domains called domain cassettes (DCs) was described^27^.

Epidemiological data suggest that the risk of severe disease declines more rapidly than mild malaria as children grow older^28^,^29^. This more rapid acquisition of immunity to severe malaria as compared to mild malaria has been suggested to be as a result of a limitation in the diversity of important immune targets in *P. falciparum*^30^,^31^. Early serological work suggests the existence of PfEMP1 subgroups with limited diversity that are preferentially expressed by parasites causing infections in children with low immunity^31^,^32^,^33^,^34^.

Various studies have attempted to profile the *var genes* expressed by clinical *P. falciparum* isolates from children diagnosed with severe and mild malaria^35^,^36^,^37^,^38^,^39^,^40^,^41^. However, different results have been obtained from these studies. This is potentially due to differences in the methods used to measure *var* expression. Previously, we used an expressed sequence tag (EST) approach using DBLα-tag amplification and sequencing to determine the *var* expression profile of clinical isolates^38^. With this method we found that the proportion of “group A-like” *var* genes expressed by the infecting parasites was positively associated with severe malaria and negatively associated with host antibodies present at the time of infection^38^,^42^. This supports the existence of PfEMP1 subsets with limited diversity^30^,^31^,^32^. This result was consistent with other studies employing a similar EST approach^36^,^43^.

Other investigators have used real-time qPCR primers designed to quantify more directly the transcript abundance of *var* genes belonging to group A, B, and C or specific domain cassettes, relative to the expression of two metabolic genes^35^,^37^,^40^,^41^. These studies found that severe malaria is associated with the transcript quantity of group A, B and subsets of group A and B *var* genes containing domain cassette 13 and 8^35^,^37^,^40^,^41^. In one study, the transcript quantity of group C *var* genes was associated with severe malaria, particularly cerebral malaria^39^. Overall, the qPCR approach suggests that severe malaria is associated with the expression of multiple PfEMP1 subsets.

Two parasite encoded histone deacetylases commonly known as *Pfsir2a* and *Pfsir2b* have been linked to differential regulation of *var* gene expression^44^,^45^. In a recent study on clinical isolates, expression levels of Pfsir2a and Pfsir2b were associated with what was described as a “dysregulation” of *var* gene expression^41^.

Since these various studies were conducted in different laboratories and on samples from different geographical areas, it is unclear whether parasites vary considerably between populations or whether the EST and qPCR approaches are providing different kinds of information.

To help resolve apparently conflicting results obtained from different studies, we brought together qPCR and EST approaches within a single dataset. We also analysed the expression of Pfsir2a, Pfsir2b together with two markers of gametocyte commitment, Pfap2-g^46^ and Pfs16^47^.

We discuss how, despite apparent discrepancies seen previously between qPCR and EST, these approaches are highly consistent with one another but provide different kinds of information. We show that both global expression of *var* genes and expression of PfSir2a by the infecting parasite population is negatively associated with the breadth of host antibodies against the IE surface carried at the time of disease. We suggest that, in addition to switching between different PfEMP1 types, the parasite regulates the quantity of PfEMP1 on the IE globally as an additional immune evasion strategy.

## Results and Discussion

To understand how PfEMP1 expression by the infecting parasite population is modified by host immunity at the time of infection, we compared *var* gene expression profiles and parasite IE phenotypes from parasites isolated from children with malaria attending Kilifi County Hospital in Kenya. We used three approaches: 1) an EST approach^37^,^38^,^40^ that gives low resolution information about the proportion of expressed *var* sequence tags sampled from each isolate falling in broadly defined *var* classes,“cys2” and a subset of cys2 called “Group A-like”^48^. 2) We used qPCR to estimate what we will call the “transcript quantity” of each of several *var* gene classes relative to two housekeeping genes^40^ (primers used are listed in Table S1). 3) As an equivalent measure of the EST from this qPCR data, we obtained what we will call the “proportional expression” of each *var* gene subset by calculating expression of each *var* gene class as a proportion of the measured total detectable *var* transcript.

### qPCR and EST approaches are consistent

The following observations demonstrated that qPCR and EST approaches to measuring *var* gene expression are consistent.

**Figure 1:**
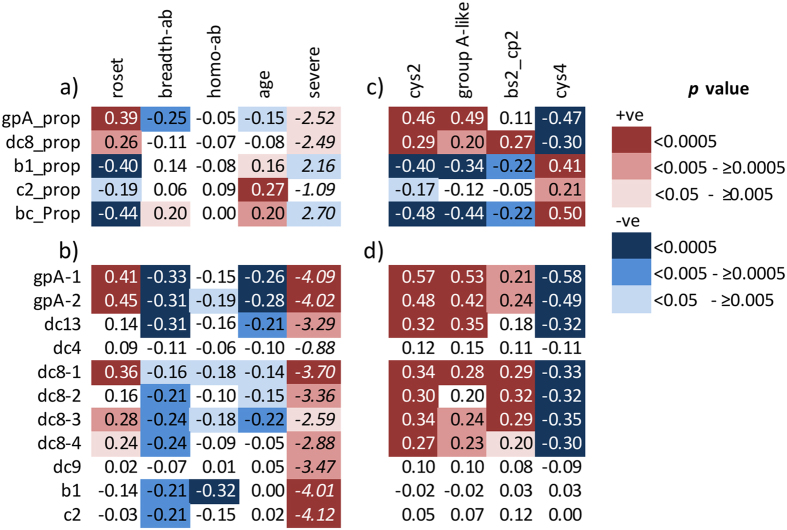
Comparisons of qPCR and EST methods of *var* expression analysis. Shown are Spearman’s rank correlation coefficients (rho) or the Mann-Whitney U test’s score (z). Mann-Whitney U test was used to test the difference between severe and non-severe malaria (negative z indicates positive association). Depth of the background shading represents the p-value as indicated in the key. Red background indicates positive associations and blue background indicate negative associations. Increasing statistical significance is shown as deeper colour. (**a**) Associations between the “proportional expression” of broad classes of *var* calculated from qPCR and parasite surface phenotype; roset (rosetting), heterologous (breadth-ab) and homologous (hom-ab) antibodies present at the time of disease, age of the children (age) and severity of the disease (severe). breadth-ab = a measure of the breadth of reactivity of each child’s antibodies present at the time of disease against infected erythrocytes of 8 heterologous *P. falciparum* clinical isolates, Homo-Ab = Antibodies against each child’s own parasite isolate. gpA_prop, dc8_prop, b1_prop and c1_prop = “proportional expression” of group A, dc8 , group B (b1) and C (c2) *var* genes respectively calculated from qPCR data. bc_prop = “proportional expression” of group B and C *var* genes together i.e bc_prop = (b1 + c2)/sum *var* transcript (see method section). (**b)** The relationship between “transcript quantity” of *var* subclasses determined using qPCR with parasite surface phenotype (rosetting), heterologous and homologous antibodies present at the time of disease, age and disease severity (as for (**a**)). Names of the primers listed in Table S1 were used to represent the “transcript quantity” of the different *var* subclasses. (**c**) Associations between the “proportional expressions” of broad classes of *var* genes calculated from qPCR data to equivalent EST proportions calculated from expressed sequence tag (EST). bs2_cp2 is a subset of cys2 that may include group B *var* genes^63^. (**d**) Associations between “transcript quantity” determined with the primers listed in Table S1 by qPCR and EST proportions. Group B (b1) and C (c2) transcript quantity did not show the expected negative association with group A-like or positive associations with cys4. N=191 except for roset and homo-ab where N=121.

First, in line with previous studies^42^,^49^, both the proportional expression (gpA_prop) and the transcript quantity of group A *var* genes (gpA1 & gpA2) were positively associated with rosetting (Fig. 1a and Fig. 1b respectively).

**Figure 2:**
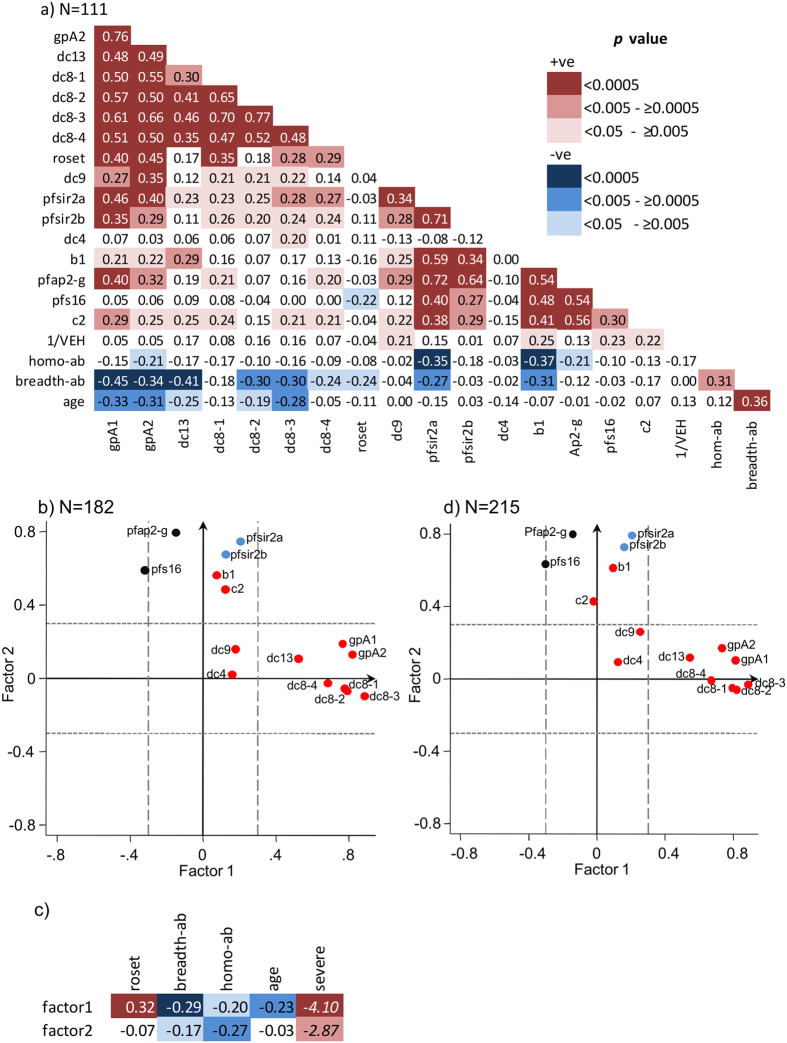
IE surface antibodies associated with global regulation of *var* gene expression. (**a**) Correlation matrix based on Spearman’s rank correlation coefficient test. Shown is the rho value and the background shading was done as described in Fig. 1. N = 111. (**b**) Principal component factor analysis of *var* expression (clinical cases only). Red; transcript quantity of the *var* subclasses represented using the names of the primers (Table S1), Light blue; PfSIR2 expression (Pfsir2a, Pfsir2b), Black; markers of gametocytogenesis (Pfap-2g, Pfs16). Factor loadings above or below 0.3 were considered significant (dashed lines). N = 182 (**c**) The relationship between predicted factor scores of factor1 and factor2 with rosetting, breadth of host antibodies, host age, and severity of the disease (N=182 except for roset and homo-ab where N=121). Shown is rho and z score derived from Spearman’s rank correlation and Mann-Whitney U tests respectively. Background colouring was done in accordance with the p-value as described in Figs 1 and 2 legends. (**d)** Same as Fig. 2b except N = 215 and includes data of parasites from 33 children with asymptomatic infection.

Second, proportional expression estimated through qPCR was highly correlated with equivalent proportions estimated by EST sequencing (Fig. 1c). Specifically, 1) proportional expression of group A *var* genes (gpA_prop) was positively correlated with group A-like expression measured using EST (Figs. 1c) and 2) proportional expression of group B and C *var* genes (b1_prop, c2_prop, and bc_prop) were negatively associated with group A-like expression measured using EST (Fig. 1c).

However, such consistency was not observed when qPCR estimates of transcript quantity of individual *var* classes were compared with proportional expression estimated through EST (Fig. 1d). Though transcript quantity of group A *var* genes was positively associated with group A-like expression estimated using EST, transcript quantity of group B and C *var* did not show expected negative associations with group A-like *var* or positive associations with cys4 (i.e. non-group A-like) *var* tags estimated using EST (Fig. 1d).

When the expression of different *var* subsets is calculated as a proportion of the total *var* transcript we implicitly make an assumption that individual parasites from different clinical isolates express equal total quantities of *var* transcript and simply make a choice in which *var* genes to express in a mutually exclusive manner. In this scenario, the difference in *var* expression patterns observed between isolates will be mainly due to differences in the proportion of parasites in the population expressing each *var* subset. If this had been the case, we would expect the transcript quantity of broad subsets of *var* within the bulk parasite population to be negatively correlated with one another. However, there was no evidence for such a relationship (see gpA1 vs b1 or c2, Fig. 2a). If expressions of different classes of *var* genes do not show clear negative associations with one another, this would suggest that the overall amount of *var* transcript in the parasite population can vary between isolates as has been suggested previously^41^.

### Transcript quantity and proportional expression analyses differ because *var* gene expression varies globally

Relaxing the assumption of constant *var* expression levels between different isolates helps in the interpretation of the relationship between *var* expression and host immunity. Previous studies have shown that group A PfEMP1 are relatively conserved in comparison to non-group A PfEMP1^31^,^32^,^33^,^34^,^38^. Consistent with this, proportional expression of group A *var* (gpA_prop) estimated by qPCR was negatively associated with IE antibodies (breadth-ab) carried at the time of disease (Fig. 1a, column 2), whilst proportional expression of groups B and C together (bc_prop) are positively associated with IE antibodies (Fig. 1a, column 2). However, when transcript quantity rather than proportional expression was considered, negative associations with IE antibodies extended to most major *var* gene subsets measured (Fig. 1b, column 2). This suggests that, rather than only selecting against parasites expressing one group of *var* genes over another, antibodies carried by the host may select more globally against high *var* gene expression.

This apparent global reduction of *var* gene expression with increasing host antibodies appears to be distinct from a reduction in the diversity of *var* genes that are expressed within each isolate. We previously used Simpsons diversity index to measure the “*var* expression homogeneity, VEH” in these parasite isolates^50^. The relationship between host antibodies and expressed *var* transcript quantity were independent of VEH in a regression analysis (Table S2).

We considered other possible causes of global variation in *var* gene expression. First, it is possible that varying commitment to gametocytogenes between parasite isolates may have led to global variation in *var* gene expression. Previous studies show that commitment to gametocytogenes is associated with an altered programme of expression of PfEMP1^51^,^52^. Recent studies have further suggested that *var* expression is controlled globally by an epigenetic mechanism that is potentially linked to gametocytogenesis^53^,^54^. Second, previous studies have suggested a role for two genes Pfsir2a and Pfsir2b in the epigenetic control of *var* gene expression^44^,^45^. One recent study has suggested that epigenetic dysregulation of *var* genes may be associated with severe malaria^41^.

We therefore explored the expression of *var* in relation to two genes involved in the epigenetic regulation of *var* genes, Pfsir2a and Pfsir2b^41^,^44^,^45^, two involved in early gametocyte differentiation, Pfap2-g^46^ and Pfs16^47^, and host IE surface antibodies carried at the time of disease. Pfsir2a was positively associated with the *var* transcript quantity amplified with the primers gpA1, gpA2, dc8-2, dc8-2, dc8-3, dc9, b1 and c2, and markers of gametocyte commitment (Pfap2-g and Pfs16) (Fig. 2a).

IE antibodies carried at the time of disease (breadth-ab) were negatively associated with Pfsir2a expression (p = 0.003, Fig. 2a) but not with Pfsir2b, Pfap2-g or Pfs16 expression. Previously, higher body temperature was suggested to positively influence Pfsir2 expression^41^. We found that body temperature shows marginal positive associations with Pfsir2 expression (Pfsir2a; rho = 0.18, p = 0.05, Pfsir2b; rho = 0.18, p = 0.05, N = 121). However when body temperature and IE surface antibody was used as explanatory variables in a regression analysis predicting Pfsir2a, only IE surface antibodies showed independent association with Pfsir2a expression (Table S3). Taken together these data support the idea that antibodies may play a role in negatively selecting parasites *in vivo* according to their broadly defined epigenetic state and suggests that this is independent of gametocyte commitment^55^.

### Principal factor analysis

To explore these relationships further we used principal factor analysis to visualize measures of *var* gene expression in relation to the expression of genes related to the control of *var* gene expression; Pfsir2a and Pfsir2b, and early markers of gametocyte differentiation; Pfap2-g and Pfs16.

**Figure 3:**
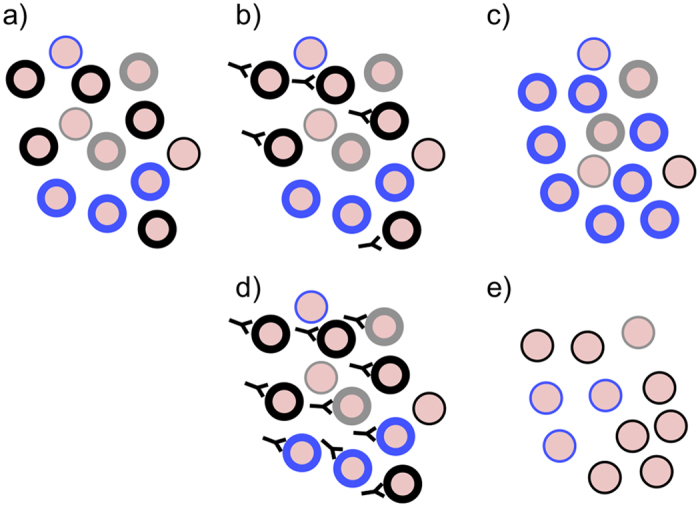
A hypothetical model for immunity dependent regulation of PfEMP1 expression. The black, blue and grey circles represent PfEMP1 variants expressed on the surface of the infected erythrocytes. (**a–c**) represents the response of parasites to partial immunity; (**a**) shows a hypothetical PfEMP1 expression profile within a naïve host where multiple PfEMP1 are expressed at high level to explore the host cytoadhesive space. Dominant variants are those which have a growth advantage due to their optimal cytoadhesion ability (**b**) antibody are raised first against the dominant variants (in this case the black type) (**c**) another variant (blue) previously expressed at lower frequency dominates the population. (**d**,**e**) represents the response of the parasite population in an individual carrying antibodies to a broad range of variants; (**d**) the pre-existing naturally acquired antibody clears all variants carrying high levels of PfEMP1, (**e**) A population of parasites expressing a previously recognised PfEMP1 but expressed at low levels of PfEMP1 survive and establish chronic infections.

Two major factors were identified in the dataset. The majority of the variables were significantly associated with one of these factors (Table S4). The different variables in the dataset were plotted in Fig. 3a in relation to each factor. Factor 1 accounts for approximately 65.84% of the variation in the data and correlates with expression of group A, DC8 and DC13 *var* genes (Fig. 2b and Table S4). Factor 2, which accounts for a further 28.18% of the variation in the data was associated with expression of group B and C *var* genes, epigenetic regulator genes; Pfsir2a and Pfsir2b, and markers of sexual commitment; Pfap2-g and Pfs16 (Fig. 2b and Table S4). Group B *var* genes, and Pfap2-g clustered with Pfsir2 and were associated with factor 2 (Table S4). This raises the possibility that expression of group B *var* genes and Pfap2-g may be linked together through a shared epigenetic mechanism as suggested by their clustering with Pfsir2.

To explore this further, we tested the associations of the predicted factor scores (see method) with rosetting, breadth of host antibodies present at time of disease, host age, and severity of the disease. As expected factor1 was associated with rosetting (p = 0.0001, Fig. 2c), however, both factor 1 and 2 were negatively associated with breadth of recognition by host antibodies present at the time of disease and positively associated with severity of disease (Fig. 2c). This is consistent with the idea that antibodies carried by the host may select globally against high *var* gene expression.

Parasites that establish chronic infections need to survive in the presence of antibodies that have been accumulated prior to infection or developed during the course of the infection. In contrast, as a result of mosquito abundance, periods of high transmission lead to opportunities to infect naïve hosts or those that have not previously encountered that particular parasite genotype and carry low levels of antibody. The apparent global association of *var* gene expression with host antibodies described here may provide a simple external control for adaptation to these two essential phases in the parasite life cycle. To test this we extended this analysis to include *var* gene expression of parasites from asymptomatic individuals who carried infections towards the end of the low transmission season. The result of this extended data is consistent with that of the smaller dataset and clusters into two factors (Fig. 2d and Table S5). The predicted scores of both factors were negatively associated with breadth of host antibodies (factor 1; rho = −0.4, p < 0.0001, factor2; rho = −0.33, p < 0.0001, Spearman’s correlation test) and asymptomatic infection (factor1; z = 5.31, p < 0.0001, factor2; z = 6.7, p < 0.0001, Mann-Whitney U test). This result raises the possibility that parasites causing asymptomatic infections under conditions of high levels of circulating antibodies may exhibit lower *var* gene expression (Fig. S1). This may be part of a mechanism to avoid immune surveillance and promote chronic infection. Despite the clustering of Pfap2-g and Pfs16 expression with factor 2 , there was no evidence for a direct correlation between IE antibody breadth and expression of Pfap2-g (Fig. 2a). This does not support a direct modulation of gametocyte commitment by antibodies. It is none the less possible that our simple measure of the breadth of IE antibodies may not fully capture the immune selection pressure experienced by the parasite population at the time of infection.

To explore this further we considered “homologous” IE antibodies tested against cultured parasites from each child as a more direct measure of the immune pressure experienced by the infecting parasite population. There was a similar negative correlation between homologous IE antibodies and Pfsir2a and a marginal negative correlation between homologous IE antibodies and Pfap2-g expression (Fig. 2a). Determining whether antibody responses to the infecting parasite population play a direct role in selecting parasites that have reduced commitment to gametocytes will require a more extensive examination of the epigenetic state of cells that escape host antibodies.

In summary, the relatively independent expression of broad classes of *var* genes described here is consistent with a model (Fig. 3) that incorporates an extended parasite bet-hedging strategy in the face of host antibodies, in which *var* expression varies both in type and expression level. Such a strategy would potentially provide the parasite population with a simple means to explore the host cytoadhesive space by simultaneously expressing multiple PfEMP1 subsets at high quantities when infecting naïve hosts^56^. As part of a form of “balancing act” between 1) retaining the cytoadhesion function of IE and 2) immune evasion, expression of low quantities of PfEMP1 may also provide means for extending within-host parasite survival when host antibodies against the infecting isolates are high. This might explain why asymptomatic infection often express group A-like PfEMP1^50^ but at low quantity (Fig.S1). This simple strategy driven by host antibodies may operate both among clinical infections of children and in the establishment of chronic infections. This model would potentially clarify the possible roles of other multigene families known to be expressed on the surface of IE and that undergo antigenic variation. Pfsir2a is also involved in the regulation of rifin genes expression^45^. In future experiments it would be interesting to determine whether expression of rifins and stevor antigens are altered in parasites that express lower levels of PfEMP1 on the surface.

## Material and Methods

### Clinical characteristics of the patients

Patient samples used in this study were described in previous publications^38^,^42^,^50^,^57^. The children presented to Kilifi County Hospital with severe and non-severe malaria. Severe malaria is defined as a hospital admission with impaired consciousness (Blantyre coma score <4 in patients under 8 months old; <5 in patients ≥8months old)^58^, respiratory distress (deep “kussmaul” pattern of breathing)^59^ or severe malarial anemia (hemoglobin <5g/dl). Non-severe cases include children admitted to hospital or attending out patient department who do not present with any of the three severe syndromes described above. Asymptomatic cases are children that were slides positive for *P. falciparum* but no clinical signs before the beginning of malaria season. The samples used in this study (N = 215, severe = 85, non-severe = 97, asymptomatic = 33) are restricted to those for which 1) sufficient sample material remained for further study 2) we previously obtained *var* expression data using the dblα-tag or expressed sequenced tags (EST) analysis method and 3) a measure had been previously made of the breadth of IE surface antibodies response present at the time of disease^38^,^42^. These samples were collected between 2003–2007.

### Ethics statement

Ethical approval for this study was obtained from Kenya Medical Research Institute (KEMRI) Ethical Review Committee (under SSC 1131), and written informed consent was obtained from parents/guardians of the study participants. The study methods were carried out in accordance with the approved guidelines.

### RNA and cDNA preparations

RNA and cDNA preparation of the samples used in this study was described previously^38^.

### Rosetting and IE surface antibody

Rosette frequency and IE surface antibody data was obtained and published previously^42^. Rosetting data was described in ref. 42. IE surface antibody data was described in refs 38,42. Homologous antibody levels were also measured^50^ whereby each child’s plasma was tested against that child’s own infecting parasite matured *in vitro* to mid to late trophozoite stage. These data was previously published in ref. 50.

### *Var* expression analysis

#### a) Expressed sequence tag (EST) sequencing approach

This data is previously published in ref. 38. Degenerate primers were used to PCR amplify a region within the DBLα-domain that is highly conserved in the majority of *var* genes, cloned into TOPO®-TA cloning vector, transformed into *E. coli*, and up to 96 colonies from each sample sequenced. The sequences were classified as described in^38^,^42^ and counted for each sample. EST proportional expression scores were obtained by counting the proportion of sequences within each sample that fell within each of the sequence groups. Group A-like tags were defined as those that had two cysteines (cys2) and carried one of a set of sequence blocks^38^.

#### *b) Var* transcript quantification using RT-PCR

Recently, 23 conserved domain cassettes (DCs) have been identified in PfEMP1^27^. Primers targeting these domain cassettes were subsequently developed and used to quantify their expression in parasites from children with severe and non-severe malaria^40^. For the present study, we selected a subset of these primers that showed differential expression between severe and non-severe malaria. These included four primers targeting DC8 (named dc8-1, dc8-2, dc8-3, dc8-4), one primer for each of DC13 (dc13), DC4 (dc4), and DC9 (dc9), two primers targeting the majority of group A *var* genes (gpA1 and gpA2) (Table S1). We also quantified the expression of group B (b1) and C (c2) *var* genes using primers described in^37^ targeting the 5′-untranslated region of the *var* classes (Table S1). In addition we quantified the expression of two genes involved in epigenetic control of *var* gene expression, Pfsir2a and Pfsir2b, and two markers of early gametocytes commitment, Pfap2-g^46^ and Pfs16^47^. We chose two housekeeping genes, Seryl tRNA synthetase and Fructose bisphosphate aldolase^40^,^60^,^61^ to use for relative quantification of the expressed *var* genes. We included dc9 targeting primer on the basis that dc9 was previously shown to be up-regulated in non-severe compared to severe malaria cases^40^. Before real-time PCR was carried out, the amplification efficiency of the primers was determined by generation of standard curves over 5 logs (100ng to 10pg of IT gDNA). The primers all had above 90% amplification efficiency. For the real time quantitative PCR, the PCR reaction and cycling conditions were carried out as described in^40^ with the Applied Biosystems 7500 Real-time PCR system. We set the cycle threshold (Ct) at 0.025. Controls with no template were included at the end of each batch of 22 samples per primer and the melt-curves analysed for non-specific amplification. We used genomic DNA from the IT4 laboratory parasite line at 10ng/μl as a standard sample included in all plates because we were able to successfully amplify this line using all the primers used in this study. To estimate the “transcript quantity” the ∆∆ct relative quantification method was used to calculate the arbitrary transcript unit (Tu_s_) using the formula (Tu_s_ = 2^(5−∆∆ct)^). However, we also estimated “proportional expression” of the transcripts within each sample (see below). When calculating proportional expression from qPCR the ∆∆ct was not appropriate and we instead used Tu_s_ calculated as described in Lavstsen *et al*.^40^ i.e. 2^(5−∆ct)^. We assigned a zero Tu_s_ value if a reaction did not result in detectable amplification after 40 cycles of amplification, i.e if the Ct value was undetermined. We excluded from the analysis if amplification of either seryl tRNA synthetase or fructose biphosphate aldose could not be obtained.

### Statistical analysis

To calculate the “proportional expression” of each *var* gene subset, we summed the transcript quantity of all the *var* subset analysed using the primers listed in table S1 (sum *var* transcript). The proportion contributed by each subset was then calculated. Since expression of group A and DC8 *var* subsets were each quantified using four different primers (Table S1), the median transcript quantity measured by these sets of group A and DC8 targeting primers was used to represent group A and DC8 respectively in the calculation of sum *var* transcript quantity and proportions. The sum of the transcript quantity of the *var* subsets was first obtained as follows; sum *var* transcript = group A_median + dc8_median + b1 + c2. The proportion of each subset was then calculated as follows; gpA_prop = group A_median/sum *var* transcript, dc8_prop = dc8_median/sum *var* transcript, b1_prop = b1/sum *var* transcript, c2_prop = c2/sum *var* transcript and bc_prop = (b1 + c2)/sum *var* transcript. Correlations between variables were evaluated using Spearman’s rank correlation coefficient or the 2-sample Wilcoxon rank-sum (Mann-Whitney) test.

### Principal factor analysis

To visualize the relationships between the multiple variables and identify potential clusters of variables, we used principal factor analysis. We first checked whether our variables are factorable. We examined the factorability of the data using Kaiser-Meyer-Olkin (KMO) tests. KMO tests the adequacy of the data for factorial analysis and KMO above 0.6 is considered adequate^62^. To optimize the factor loadings (the relationship of each variable to the factors) and obtain simple structures where the variables are significantly associated with one of the factors and poorly on the others, the loadings were rotated using the *promax* rotation method and loading >0.3 or <−0.3 was considered significant. We used promax because it resulted in the best defined structure with the majority of variables loading above 0.3 or below −0.3.

We used the Stata command “predict” to generate predicted factor scores for each individual. We then tested the relationship between rosetting, breadth of host antibodies, severity of the disease, asymptomatic infection and the factor scores using Spearman’s rank correlation coefficient and Mann-Whitney U tests.

To eliminate zeros from the Tu_s_ data we added 0.1 to all values and then log transformed the resultant Tu_s_ values to normalize the distribution of the data before use in factor analysis. All statistical tests were performed using Stata software version 13 (Stata Corp, College Station, Tx).

## Additional Information

**How to cite this article**: Abdi, A. I. *et al*. Global selection of *Plasmodium falciparum* virulence antigen expression by host antibodies. *Sci. Rep.* **6**, 19882; doi: 10.1038/srep19882 (2016).

## Supplementary Material

Supplementary Information
