# Supplementary material for: Global selection of Plasmodium falciparum virulence antigen expression by host antibodies
Source: Sci Rep. 2016 Jan 25;6:19882. doi: 10.1038/srep19882 (PMC4726288; doi:10.1038/srep19882)
Supplement: Supplementary Information [file srep19882-s1.pdf]

Supplementary information

Global selection of *Plasmodium falciparum* virulence antigen expression by host antibodies

Abdirahman I. Abdi[1, 2*], George M. Warimwe[4'5], Michelle K. Muthui[1], Cheryl A. Kivisi[1], , Esther W. Kiragu[1], Gregory W. Fegan[1, 3] , Peter C. Bull*[1, 3]

Figure-S1: *var* expression quantity in severe, non-severe, and asymptomatic infections

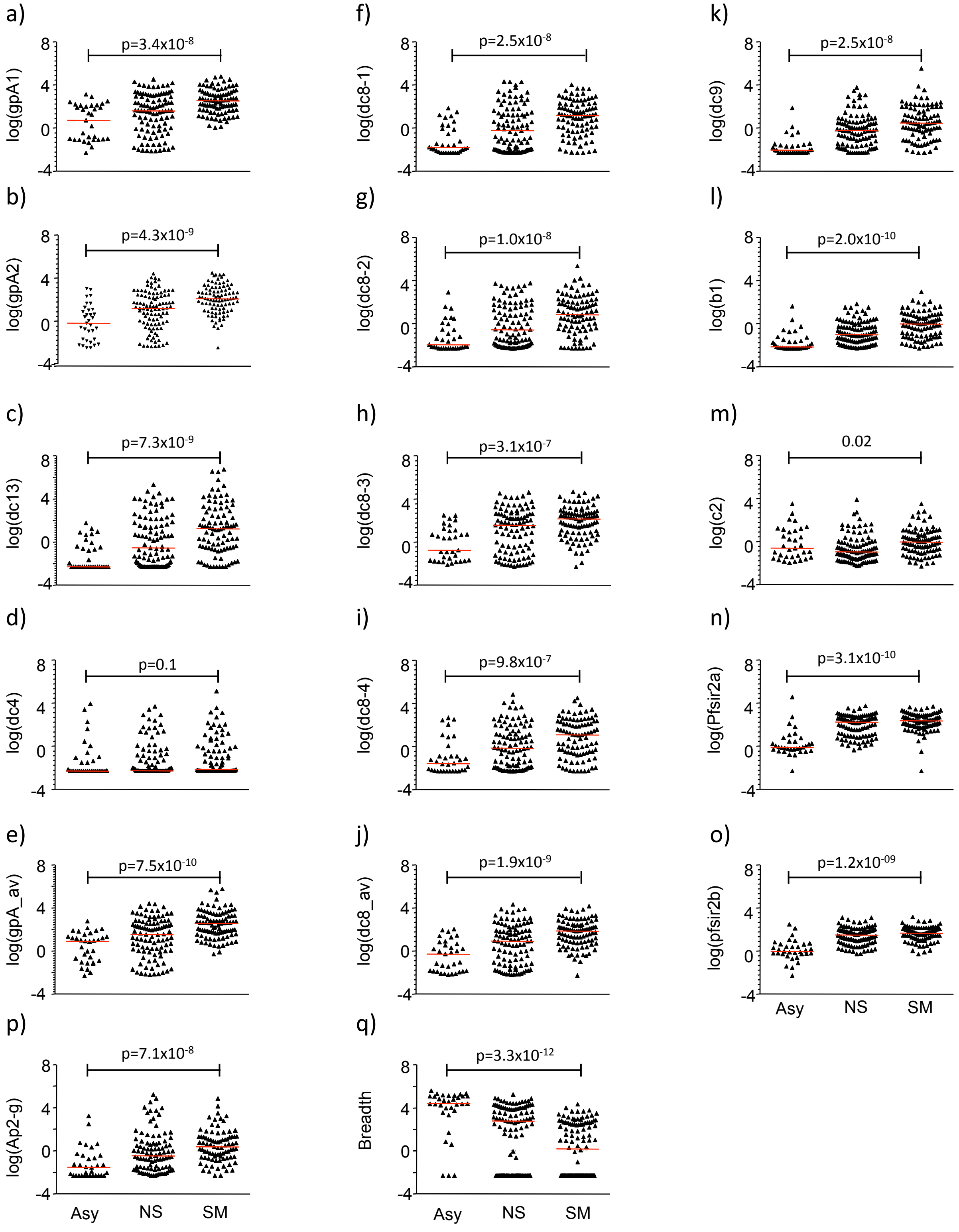

Figure-S1: ***var* transcript quantity expressed by parasites from children with severe (SM), non-severe (NS) and asymptomatic (asy) infections**. Shown on the y-axis is log transformed arbitrary transcript quantity obtained with the primers listed in Table S1. a-e represent transcript obtained with group A targeting primers where e is the median transcript quantity of group A var genes. g-j represent transcript obtained with dc8 targeting primers where J is the median transcript quantity obtained with the dc8 targeting primers. Included in the analysis is also expression of Pfsir2a(n) , Pfsir2b(o), and Pfap2-g(p). We have also shown the breadth of antibodies against IE surface antigens circulating during the time of infection(q). The red horizontal bar represent the median. p-value was calculated using Cuzick's test for trend. All except dc4 showed significant trend.

Table-S1: The list of primers used in this study and their targets

	Primer name	Name given	Reference	Targets
1	dbla_not_var3	gpA1	[1]	Majority of group A *vars*
2	dbla2/a1.1/2/4/7	gpA2	[1]	Mainly group A but also DC8
3	cidra1.4	dc13	[1]	group A subset containing domain cassette 13
4	cidra1.6	dc4	[1]	Group A containing cidra1.6
5	cidra1.1	dc8-1	[1]	Group B subset containing domain cassette 8
6	dbla_cidra	dc8-2	[1]	Group B subset containing domain cassette 8
7	dblb12&dblb3&5	dc8-3	[1]	Group B subset containing domain cassette 8
8	dblg4/6	dc8-4	[1]	Group B subset containing domain cassette 8
9	dblz4	dc9	[1]	Group B subset containing domain cassette 9
10	upsB1	b1	[2]	5' sequence of majority of group B
11	upsC2	c2	[2]	5' sequence of majority of group C
12	PfAP2-g	Pfap2-g	[3]	within coding region
13	Pfsir2a<br>Fwd:CCTTAACAGGGTCAGGTACA<br>Rev:CCAAAAACCCCATATAGTTCCA	Pfsir2a	This study	within coding region
15	Pfsir2b<br>Fwd:AGGGCCACTAGGTGAAGAAG<br>Rev:GTTGATATGCCAGCACCTGA	Pfsir2b	This study	within coding region
16	Seryl-tRNA_synthatase		[4]	
17	Fructose_biphosphase aldolase		[4]	

"Primer name" is the name of the primer in the original study (see reference column), "Name given" is the name given to the primer in this study. Primer gpA2 targets group A *var* genes containing dbla1.1/2/4/7 but also can amplify the dbla2 of dc8.

Table-S2: The association between the breadth of host antibody during time of disease and *var* transcript quantity

Models	Variable	Group A	DC8	DC9	Group B	Group C
1 a, b, c, d, e	breadth-ab	-0.23(-0.30, -0.15)<br>*3.42603e-09*	-0.18(-0.26, -0.10)<br>*0.00002*	-0.11(-0.18, -.03)<br>*0.005*	-0.14(-0.19, -0.08)<br>*1.19764e-06*	-0.04(-0.10, 0.02)<br>*0.2*
2 a, b, c, d, e	VEH	0.63(0.35, 0.90)<br>*0.00001*	0.64(0.34, 0.93)<br>*0.00004*	0.87(0.61, 1.12)<br>*1.59854e-10*	0.52(0.33, 0.72)<br>3.72100e-07	0.31(0.09, 0.53)<br>0.006
3 a, b, c, d, e	breadth-ab	-0.21(-0.28, -0.14)<br>*2.58643e-08*	-0.16(-0.24, -0.08)<br>*0.0001*	-0.08(-0.15, -0.01)<br>*0.028*	-0.12(-0.17, -0.07)<br>*4.9 e-06*	-0.03(-0.09,0.02)<br>*0.2*
	VEH	0.52(0.26, 0.78)<br>*0.0001*	0.56(0.27, 0.85)<br>*0.0002*	0.8(0.55,1.06)<br>*2.4e-09*	0.45(0.26,0.64)<br>*5.16 e-06*	0.29(0.07, 0.51)<br>*0.009*

The table shows the regression coefficient, 95%CI, and *p* values obtained from a set of regression analysis models predicting expression of either group A (models 1a, 2a, 3a), DC8 (models 1b, 2b, 3b), dc9 (models 1c, 2c, 3c), group B (b1) (models 1d, 2d, 3d) or group C (c2) (models 1e, 2e, 3e) *var* genes. Breadth of antibody recognition (models 1 a, b, c, d, e), *var* expression homogeneity (VEH)(models 2 a, b, c, d, e) or combination of the two variables were used as independent variables. Except for expression of group C (c2) *var* genes, the association of breadth of antibody (breadth-ab) with *var* gene expression is independent of VEH. In this regression analysis, breadth of antibody is not significantly associated with expression of group C (c2) *var* genes even when used as the sole explanatory variable. breadth-ab = the median of each child's plasma reactivity against 8 heterologous parasites measured by flow cytometer. Group A= The median transcript quantity obtained with the primers gpA1, gpA2 & dc13 while DC8= the median transcript quantity obtained with the primers dc8-1, dc8-2, dc8-3, dc8-4, Table S1. N=215

Table-S3: The relationship between body temperatures, breadth of antibody and Pfsir2a expression

Model		Pfsir2a Coeff(95%CI)	P value
1	*Temp (°C)*	*3.4(-1.72, 8.44)*	*0.193*
2	*breadth-ab*	*-0.10(-0.16, -0.04)*	*0.001*
3	*Temp (°C)*	*2.98(-1.89, 7.86)*	*0.2*
	breadth-ab	*-0.10(-0.16, -0.04)*	*0.001*

Shown is regression coefficient, 95%CI, and p value of three regression models predicting expression of pfsir2a using as explanatory variables; 1) admission body temperature (Temp) and 2) breadth of antibody against the surface of infected erythrocytes present at the time of disease).
breadth-ab was log transformed before use in regression analysis. Only sample with available antibody and body temperature data were included in this analysis N=121.

Table-S4:Oblique Promax rotated factor loadings

Variables	factor1	factor2	uniqueness
dc8-1	***0.78***	-0.06	0.41
dc8-2	***0.79***	-0.07	0.40
dc8-3	***0.89***	-0.10	0.26
dc8-4	***0.68***	-0.03	0.54
dc13	***0.52***	0.11	0.68
gpA1	***0.77***	0.19	0.29
gpA2	***0.82***	0.13	0.25
dc4	*0.16*	*0.02*	0.97
dc9	0.18	*0.16*	0.92
b1	-0.08	***0.56***	0.65
c2	0.12	***0.49***	*0.71*
Pfsir2a	0.21	***0.75***	0.31
Pfsir2b	0.13	***0.67***	0.48
Pfap2-g	-0.15	***0.79***	0.41
Pfs16	-0.32	***0.59***	0.66
Eigenvalue	4.9	2.10	
Proportion	65.84%	28.18%	

The result of promax rotation of principal factor analysis is shown in the table.
Significant Loadings (> 0.3 or <-0.03) were highlighted bold. Strictly following the significance cut-off at loading >0.3 or <-.03, dc4 and dc9 are not associated with any of the two factors. Kaiser-Meyer-Olkin[5] measure of sampling adequacy was 0.85 suggesting that our data qualifies for factor analysis. N=182

Table-S5: Oblique promax rotated factor loadings

Variables	Factor1	Factor2	Uniqueness
dc8-1	**0.80**	-0.05	0.40
dc8-2	**0.82**	-0.06	0.36
dc8-3	**0.89**	-0.03	0.23
dc8-4	**0.67**	-0.01	0.55
dc13	**0.54**	0.12	0.64
gpA1	**0.74**	0.17	0.34
gpA2	**0.81**	0.10	0.26
dc4	0.12	0.09	0.97
dc9	0.26	0.26	0.82
b1	0.09	**0.61**	0.57
c2	-0.02	**0.43**	0.82
Pfs16	-0.30	**0.63**	0.65
Pfsir2a	0.2	**0.79**	0.21
Pfsir2b	0.16	**0.73**	0.36
Pfap2-g	-0.14	**0.80**	0.42
Eigenvalue	5.4	2.0	
proportions	69.83%	25.87%	

Shown are the association between the variables listed and the two factors with Eigenvalue >1. Loadings >0.3 or <-0.3 were highlighted bold. N=215, KMO=0.87 suggesting the data qualifies for use in factor analysis.

References.

[1] Lavstsen T, Turner L, Saguti F, Magistrado P, Rask TS, Jespersen JS, Wang CW, Berger SS, Baraka V, Marquard [illegible] Seguin-Orlando A, Willerslev E, Gilbert MT, Lusingu J, Theander TG: Plasmodium falciparum erythrocyte membrane [illegible] protein 1 domain cassettes 8 and 13 are associated with severe malaria in children. Proc Natl Acad Sci U S A 2012, 109:E1791-800.
[2] Rottmann M, Lavstsen T, Mugasa JP, Kaestli M, Jensen AT, Muller D, Theander T, Beck HP: Differential [illegible] of var gene groups is associated with morbidity caused by Plasmodium falciparum infection in Tanzanian children. Infect Immun 2006, 74:3904-11.
[3] Kafsack BF, Rovira-Graells N, Clark TG, Bancells C, Crowley VM, Campino SG, Williams AE, Drought LG, Kwiatkowski DP, Baker DA, Cortes A, Llinas M: A transcriptional switch underlies commitment to sexual [illegible] in malaria parasites. Nature 2014, 507:248-52.
[4] Salanti A, Staalsoe T, Lavstsen T, Jensen AT, Sowa MP, Arnot DE, Hviid L, Theander TG: Selective upregulation of [illegible] single distinctly structured var gene in chondroitin sulphate A-adhering Plasmodium falciparum involved in pregnancy-associated malaria. Mol Microbiol 2003, 49:179-91.
[5] HF K: An index of factor simplicity. Psychometrika 1974, 39:31-6.
